# Early-life phthalate mixture exposure and reproductive hormone levels during minipuberty

**DOI:** 10.3389/fendo.2026.1866609

**Published:** 2026-07-22

**Authors:** Viola Trevisani, Laura Lucaccioni, Federico Starace, Erica Passini, Angela Ferrari, Lucia Palandri, Camilla Lugli, Diego Pinetti, Enrico Tagliafico, Licia Lugli, Barbara Predieri, Lorenzo Iughetti, Elena Righi

**Affiliations:** 1 PhD program in Clinical and Experimental Medicine, Department of Biomedical, Metabolic and Neural Sciences, University of Modena and Reggio Emilia, Modena, Italy; 2 Pediatric Unit, Department of Medical and Surgical Sciences of the Mothers, Children and Adults, University of Modena and Reggio Emilia, Modena, Italy; 3 Public Health Unit, Department of Biomedical, Metabolic and Neural Sciences, University of Modena and Reggio Emilia, Modena, Italy; 4 Post-Graduate School of Pediatrics, Department of Medical and Surgical Sciences of the Mothers, Children and Adults, University of Modena and Reggio Emilia, Modena, Italy; 5 Interdepartmental Centre for Large Instruments (CIGS), University of Modena and Reggio Emilia, Modena, Italy; 6 Diagnostic Hematology and Clinical Genomics Unit, Department of Medical and Surgical Sciences of the Mothers, Children and Adults, University of Modena and Reggio Emilia, Modena, Italy; 7 Neonatal Intensive Care Unit, Department of Medical and Surgical Sciences of the Mothers, Children and Adults, University of Modena and Reggio Emilia, Modena, Italy

**Keywords:** EDCs, endocrine-disrupting chemicals, minipuberty, mixture exposure, phthalates, urinary gonadotropins

## Abstract

**Background:**

Phthalates (PAEs) are ubiquitous endocrine-disrupting chemicals (EDCs) with well-documented prenatal effects, while their impact during early postnatal life remains less understood. Minipuberty, a transient activation of the hypothalamic-pituitary-gonadal (HPG) axis in early infancy, represents a critical window of endocrine programming. This study aimed to investigate the association between early-life exposure to PAEs and urinary reproductive hormone levels during minipuberty in healthy term-born infants.

**Methods:**

This longitudinal study included infants from the Modena birth cohort with repeated urine sampling at birth (T0), 3 (T3), and 6 months (T6). Urinary concentrations of PAE metabolites (MMP, MEP, MnBP, MBzP, ΣDEHPm) and urine reproductive hormones (uLH, uFSH, uT and uE) were measured. Associations were assessed using sex-stratified Spearman correlations, single-pollutant linear regression models, and mixture approaches, including Weighted Quantile Sum (WQS) regression and quantile g-computation (qgcomp).

**Results:**

PAE metabolites were evaluated in 162 infants and detected in nearly all samples, confirming widespread exposure. Consistent positive associations emerged between PAE exposure and urinary hormone levels. In males, increasing levels of metabolites were significantly associated particularly with increased uFSH and uT, at all timepoints. In females, associations were more evident at T3 and T6, mainly involving uFSH and uE. Mixture analyses confirmed these findings and identified MnBP, MBzP, and ΣDEHPm as the main contributors. Results were consistent across methods, highlighting a mixture association beside an isolated compound-specific findings.

**Conclusion:**

Early-life exposure to PAE mixtures is associated with sex-specific alterations in reproductive hormone levels during minipuberty. These findings highlight minipuberty as a sensitive window for endocrine disruption and underscore the importance of considering chemical mixtures in environmental health research. However, further studies are needed to determine whether these early hormonal perturbations may have implications for long-term reproductive development and health.

## 1 Introduction

Phthalates (PAEs) are non-persistent chemicals widely used as solvents or plasticizers in plastics and numerous consumer products (1). Due to their weak binding to polymer matrices, they are readily released into the environment, resulting in ubiquitous human exposure through ingestion, inhalation, and dermal contact. Although rapidly metabolized and excreted primarily in urine within hours or days (2–4), continuous exposure results in a persistent internal burden. Some PAEs are recognized endocrine-disrupting chemicals (EDCs), interfering with hormonal homeostasis even at low doses and stringer regulations in their use has been recently implemented in many countries (5). Given their ubiquity, continuous exposure, and endocrine-disruption potential, understanding their impact during early-life critical windows has become a major priority in environmental and pediatric health research.

Humans are particularly vulnerable to PAEs during critical windows of development, especially during fetal life and early infancy (1, 6–12). During these periods, endocrine signals orchestrate tightly regulated processes essential for organogenesis and functional maturation. Experimental and epidemiological evidence indicates that PAEs can interfere with gonadal development, steroidogenesis, and neurodevelopment, potentially affecting growth trajectories, pubertal timing, fertility, and increasing the risk of hormone-sensitive diseases later in life (1, 2, 13–18). This framework is consistent with the developmental origins of health and disease (DOHaD) paradigm, whereby early-life environmental exposures can induce long-lasting endocrine and metabolic alterations (19). In particular, adverse effects of prenatal exposure to endocrine-disrupting PAEs are now well documented (20–25). Blood-based studies measuring serum or plasma PAE concentrations in pregnant women have demonstrated associations between circulating PAE burden and clinically relevant perinatal outcomes, including preterm birth (6, 26, 27). However, the postnatal period remains comparatively underexplored, despite representing a phase of rapid endocrine adaptation.

The transient postnatal activation of the hypothalamic–pituitary–gonadal (HPG) axis during early infancy, known as minipuberty, represents a critical postnatal developmental window (28–30). This physiological surge in gonadotropins and sex steroids during the first months of life is essential for children, and especially for male genital development, Sertoli and Leydig cell maturation, and the establishment of future reproductive capacity (31, 32). Emerging evidence suggests that minipuberty also functions as a window of endocrine programming, where hormonal patterns may reflect and potentially predict later reproductive function (32). As a tightly regulated and transient hormonal surge, minipuberty provides a unique *in vivo* model to investigate early-life endocrine disruption in humans. The finely tuned interplay among hypothalamic, pituitary, and gonadal hormones makes this period particularly sensitive to endocrine disruption (1, 2, 33, 34). Consequently, environmental exposures during minipuberty could have both immediate hormonal effects and longer-term consequences for reproductive health.

As the impact of prenatal PAE exposure is well documented, recent attention has shifted toward early postnatal exposure as a potential disruptor of minipuberty. Evidence addressing this specific window remains limited, but available studies indicate that early postnatal exposure -assessed through breast milk or infant urinary concentrations- is associated with measurable alterations in reproductive hormone profiles, including changes in gonadotropins and testosterone/LH ratio (35, 36).

Despite these preliminary findings, further research is needed to clarify the relationship between perinatal and early postnatal PAEs exposure, taking into account also a mixture effect, and hormonal dynamics during minipuberty, particularly in cohorts of healthy, full-term infants. Accordingly, the present study aims to evaluate the association between urinary PAEs metabolite concentrations and urinary reproductive hormone levels during minipuberty in a cohort of healthy term-born children. Specifically, we apply a longitudinal design and a multi-exposure analytical framework to investigate how PAE mixtures influence hormonal levels during this critical developmental window. By focusing on a physiologically normal population, the study seeks to identify subtle endocrine variations within the expected range of minipubertal activation, thereby improving our understanding of environmental influences on early HPG axis function.

## 2 Materials and methods

### 2.1 Study population

**Figure 1:**
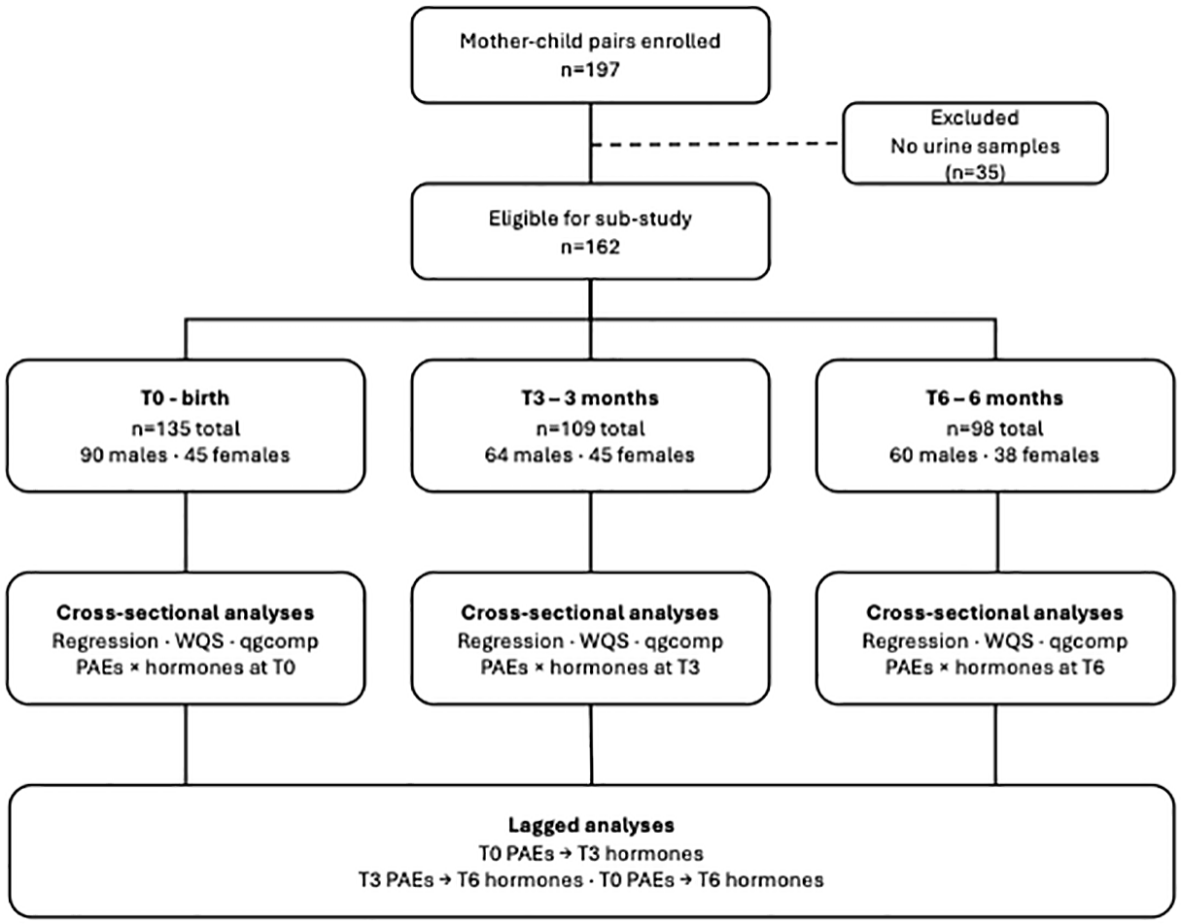
Flow diagram of participant selection and urine sample availability.

The present study analyzes data from the Modena cohort study, a prospective birth-cohort study, which aims to assess perinatal and postnatal exposure to PAEs, and the association between this exposure and hormonal, anthropometric and neurocognitive development in children aged 0–3 year. The study started in 2019, and the enrollment ended in October 2020. Newborns whose mothers were of legal age (≥18 years old) at delivery and had a singleton pregnancy were eligible to participate in the main cohort study. Other inclusion criteria based on neonatal characteristics were term delivery (37–41 weeks post menstrual age), newborns appropriate-for-gestational-age (AGA), Apgar score >7 five minutes after birth. The sample size calculation was estimated in about 200 newborns; therefore, a total of 197 mothers-child pairs were enrolled. Data presented derived from a subsample of the whole Modena cohort study; in particular, eligibility criteria were to have at least one urine sample for hormonal assessment and one for PAEs assessment from birth to 6 months. The flow diagram of participant selection and sample availability at each timepoint is presented in **Figure 1**.

### 2.2 Urine sample collection and preparation

Urine samples were collected at birth (T0), 3 months (T3) and 6 months of age (T6). Spot urine was collected in pediatric PAE free urine bags attached to the skin during the examinations. Sample collection during follow-up visits was performed by trained personnel or by caregivers instructed on correct sampling techniques both verbally and with pamphlets explaining how to collect a urine sample for their child. Once the sample was obtained, it was collected by the researchers.

Urinary dilution correction was not applied to either phthalate metabolite or reproductive hormone concentrations. For urinary hormones, creatinine correction was not used as creatinine excretion in children is subject to wide variability driven by factors beyond urine dilution, including muscle mass, age, sex, and dietary habits, and does not reliably improve the correlation between urinary and serum concentrations (37, 38). For PAE metabolites, given the rapid metabolism of PAEs and the intermittent nature of exposure, creatinine adjustment should be used cautiously as it may distort true exposure estimates (39–41).

#### 2.2.1 Hormone analysis

Each urine sample was analyzed by the Department of Laboratory Medicine and Pathological Anatomy of the University Hospital of Modena. All four urinary hormones, urinary Follicle-Stimulating Hormone (uFSH), urinary Luteinizing Hormone (uLH), urinary Testosterone (uT), and urinary Estradiol (uE), were quantified using an electrochemiluminescence immunoassay (ECLIA, Roche Diagnostics, Cobas E601).

All urinary hormone assays were performed on fresh urine samples wherever possible. When analysis on fresh urine was not feasible (e.g., late afternoon follow-up visits), samples were stored at 4 °C and analyzed within 24 hours. This approach is supported by evidence that gonadotropins, while unstable after freezing at -20 °C, can be accurately assessed after short-term storage at 4 °C with performance comparable to fresh samples.

For uFSH and uLH, the lower limit of detection (LOD) was 0.1 mIU/mL for both analytes. Intermediate precision was <4.5% and <3.7% at concentrations ranging from 0.65 to 152 mIU/mL for uFSH, and <2.2% and <1.6% at concentrations ranging from 1.3 to 123 mIU/ml for uLH. For uTs, the analytical range was 0.025–15 ng/mL, with intermediate precision <14.8% and <2.1% at concentrations ranging from 0.063 to 14 ng/ml (conversion factor: ng/mL × 3.47 = nmol/L). For uE, the analytical range was 18.4–11010 pmol/L, with intermediate precision <10.6% and <1.9% at concentrations ranging from 10.2 to 2934 pg/mL (conversion factor: pg/mL × 0.00367 = nmol/L).

We acknowledge that immunoenzymatic assays are not yet formally validated for the urinary detection of gonadotropins, testosterone, and estradiol in infant samples, and that formal matrix effect assessment was not performed. However, this limitation is shared across the field. The use of urinary hormones was chosen to minimize invasiveness in neonates and infants, and is supported by a growing body of literature demonstrating strong correlations between urinary and serum concentrations for these hormones (42–47).

#### 2.2.2 Phthalates assays

The total content (sum of free and conjugated) of 7 major metabolites of 5 of the most commonly used PAEs (**Table 1**) in urine samples collected at T0, T3 and T6 months was measured by quantitative determination by high performance liquid chromatography – mass spectrometry (UHPLC–MS), after enzymatic deconjugation and solid phase extraction (SPE) of the collected urine samples.

**Table 1:** Names, abbreviations, molar weight of investigated phthalate parents and their metabolites.

Phthalates		Abbreviation	Molar weight (g/mol)
Phthalates diester	Urine metabolites		
Di-methyl phthalate		DMP	194.18
	Mono-methyl phthalate	MMP	184.18
Di-ethyl phthalate		DEP	224.24
	Mono-ethyl phthalate	MEP	278.34
Di-n-butyl phthalate		DnBP	278.34
	Mono-n-butyl phthalate	MnBP	222.24
Buthylbenzyl phthalate		BBzP	312.36
	Mono-benzyl phthalate	MBzP	256.257
Di-(2-ethyl-hexyl) phthalate		DEHP	390.56
	Mono-(2-ethyl-hexyl) phthalate	MEHP	278.34
Mono-(2-ethyl-oxohexyl) phthalate	MEOHP	292.33
Mono-(2-ethyl-oxohexyl) phthalate	MEHHP	294.34

Analytical procedures were optimized by modifying and expanding methods published by the USA Centers for Disease Control and Prevention (CDC) Environmental Health Department (48). Sample storage, sample preparation and SPE were carried out at the Laboratory dedicated to environmental chemical analyses of the Department of Biomedical, Metabolic and Neurological Sciences of the University of Modena and Reggio Emilia (Italy); UHLC-MS/MS analyses were performed at the Interdepartmental Centre for Large Instruments of the same university.

Reagents and standards used are reported in **Supplementary Materials**. Milli-Q water was cleaned in a Millipore system (Merk Millipore) at the Interdepartmental Centre for Large Instruments (University of Modena and Reggio Emilia, Italy). All chemicals were of analytical, HPLC or MS grade and all chemicals, solutions, sampling materials and laboratory equipment were controlled for contamination of the investigated metabolites before use.

For determining calibration curve and range of linearity eleven pools of standards solutions at different and known and concentrations in the range 0.05–100 ppb (0.05; 0.1; 0.25; 0.50; 1.00; 2.50; 5.00; 10; 20; 40 and 100 ppb). The limit of detection (LOD) and the other main validation parameters of the analytical method are reported in **Supplementary Table 1**.

Aliquots (at least 1 mL) of urine samples collected as previously described were immediately transferred into glass vials and frozen at -20 °C until analyzed for PAE metabolite content. After thawing, urine samples were mixed and centrifugated (5 min at 5000 rpm/min) and, finally, aliquots of 1 mL were used. Samples were added with 10 ppb of each isotope labelled standard (Monomethyl phthalate (ring-1.2-¹³C_2_, dicarboxyl-¹³C_2_, 99%) and Mono-ethyl phthalate (ring-1.2-13C2.99%, dicarboxyl-13C2.99%)), and with 200 μL of β-glucuronidase enzyme solution (1M ammonium acetate buffer, pH 6.5) to hydrolyze the glucuronidated metabolites, vortexed for 30 seconds and then incubated for 120 minutes at 38 °C to allow the cleavage of monoester glucuronides by the enzyme. The reaction was blocked by adding 500 μL of glacial acetic acid, vortex for 10 seconds, adding 1200 μL of acetonitrile (5%) in Milli-Q water and vortex for 30 seconds.

To reduce impurities that could affect matrices and alter the sample selectivity and specificity, the samples were then extracted by solid phase extraction (SPE). SPE columns Bond Elut C18–500 mg 6 mL (Agilent Technologies, DTO, Italy), previously activated with 2 mL methanol and 2 mL Milli-Q water, were used. After SPE, 1 mL of a solution of acetic acid (0,1%) in acetonitrile was used to eluate PAE metabolites constituting the final solution for the following instrumental analyses.

Finally, PAE metabolites were detected and quantified by an UHPLC-Q-Exactive Orbitrap analysis conducted with a liquid chromatography-mass spectrometry setup. It combined an Ultimate 3000 UHPLC and a Q-Exactive Orbitrap (Thermo Fisher Scientific Waltham, MA, USA), operating in Targeted SIM/Data Dependent (t-SIM/dd - MS2) acquisition mode. The system was equipped with a heated electrospray ionization source HESI II (Thermo Fisher Scientific Waltham, MA, USA). Chromatographic separation was carried out using a Raptor ARC-18 5μm 150 x 2,1 mm ID column at 25 °C (Restek – Superchrom, Milano, Italy). Further, a Zorbax Eclipse XDB-C18 1,8 μm 50 x 4.6 mm ID column (Agilent Technologies Santa Clara, CA, USA) was placed upstream of the injection valve and used as a hold-back column. Solvent A (Milli-Q water with 0.1% acetic acid) and B (acetonitrile with 0.1% acetic acid) served as the mobile phases (gradient elution program in **Supplementary Table 2**). Before injection batch samples were diluted with mobile phase A (1:1). Autosampler temperature was set at 15 °C, the sample injection volume was 10 μL, and the flow rate was 0.30 mL/min. The Q-Exactive Orbitrap MS with a heated electrospray ionization source (HESI) operated in negative ion mode. Mass spectrometer’s operational settings were as follows: the ion spray voltage was 3200 V for (–)-ESI, 320 °C capillary temperature, 290 °C auxiliary gas heater temperature, 37 arb sheath gas flow rate, 28 arb auxiliary gas flow rate.

All batches besides urine samples included laboratory blanks (blanks containing mobile phase A and B and laboratory blanks with reagents and solvents used in SPE procedure) and eleven pools of standards solutions at different and known and concentrations in the range 0.05–100 ppb (0.05; 0.1; 0.25; 0.50; 1,00; 2.50; 5.00; 10; 20; 40 and 100 ppb) for calibration curve.

The possibility of maintaining a constant temperature of 15 °C of samples inside the autosampler allowed us to work up to even 72 hours without significant variations in linearity and intra-session reproducibility, with a relative standard deviation (RSD) <10%. Longer work sessions were not performed. Work sessions carried out even weeks apart reported an RSD always <20%.

Metabolite concentrations were expressed in ng/mL. Metabolites of DEHP were expressed as ∑DEHPm, by using equation


$$\sum DEHPm=[(\frac{UC_{m1}(\frac{ng}{mL})}{MW_{m1}(\frac{ng}{nmol})})+(\frac{UC_{m2}(\frac{ng}{mL})}{MW_{m2}(\frac{ng}{nmol})})+\ldots ]\times MW_{p}(\frac{ng}{nmol})$$

where ∑DEHPm is the summed urinary concentration of metabolites of DEHP (MEHP, MEHHP, MEOHP) expressed as the urinary concentration of parent compound; UCm1, UCm2 etc. are the urinary concentration of the individual metabolites and MWm1, MWm2 etc. indicate molecular masses of each metabolite, respectively, while MWp is the molecular mass of the parent compound.

### 2.3 Statistical methods

All analyses were conducted within an exploratory framework, aimed at generating hypotheses about the associations between PAE mixture exposure and urinary reproductive hormone levels during minipuberty.

Numeric variables, including hormone and PAE concentrations, were summarized using mean, standard deviation, median, and selected percentiles. Hormone concentrations below LOD were replaced with LOD/2 and values of PAE metabolites below LOD were imputed as LOD/√2, in accordance with standard practice.

Due to right-skewed distributions, both hormone and phthalate concentrations were natural log-transformed prior to all inferential analyses. All analyses were stratified by infant sex and sampling time, and missing data were handled using pairwise-case analysis.

Spearman’s rank correlation coefficients were firstly calculated to assess associations between hormone levels and phthalate metabolites, with correlation strength classified as weak (ρ = 0–0.39), moderate (ρ = 0.40–0.59), or strong (ρ ≥ 0.60).

Associations between individual phthalates and hormones were then further examined using linear regression models fitted separately for each phthalate–hormone pair (single-pollutant models), specified as ln(hormone) ~ ln(phthalate) + age (in days). Age at sample collection was included as the sole covariate in all models. The Benjamini–Hochberg procedure was applied within each hormone to control the false discovery rate. The use of single-pollutant linear regression models, rather than multi-pollutant approaches, was justified by moderate-to-high inter-metabolite correlations (Spearman’s ρ up to 0.75), evidence of multicollinearity (variance inflation factors > 3 in several strata), and the limited sample size.

To assess mixture associations two complementary approaches were used following the framework of Zhao et al. (49). Weighted Quantile Sum (WQS) (50) regression was used to estimate a weighted index of the exposure mixture under directional constraints. Separate models were fitted assuming positive and negative associations, using quartile-based categorization of exposures, 200 bootstrap iterations, and a 60/40 training/validation split. Component weights, constrained to sum to 1, were used to quantify the relative contribution of each metabolite.

Quantile g-computation (qgcomp) (51) was also applied, allowing for bidirectional effects within a single model without requiring data splitting. Models were fitted using 200 bootstrap samples; the overall mixture association (ψ) represents the expected change in ln(hormone) associated with a simultaneous one-quartile increase in all exposures, with exponentiated estimates interpreted on the original scale. To test for departure from linearity at the mixture level a quadratic (degree-2) quantile g-computation model, in which the squared mixture index (ψ_2_) quantifies curvature of the joint exposure–response relationship was set up.

Finally, lagged models were fitted to explore whether PAE metabolite concentrations at earlier timepoints were associated with urinary hormone levels at subsequent timepoints. Specifically, T0 PAE concentrations were used to assess T3 or T6 hormone levels, T3 PAE concentrations to evaluate T6 hormone levels. Lagged models were carried out using the same methodological approaches (single-pollutant models, WQS, and qgcomp) as the primary cross-sectional analyses, stratified by sex and adjusted for age in days.

All analyses were conducted using R (version 4.3.1; R Core Team, Vienna, Austria).

### 2.4 Ethical considerations

The study was approved by Area Vasta Emilia Nord Ethics Committee (2018/num715). Informed consent to participation and publication of the data was obtained from caregivers of all individual participants included in the study.

## 3 Results

### 3.1 Study population

One hundred and sixty-two neonates (98 males, 60.5%) were found eligible for this sub-study. all born at term (39.39±1.03 weeks of gestation), AGA, and with an Apgar score >7 at 5 minutes. At baseline (T0) 135 infants (90 males, 66.7%) were evaluated, showing good general conditions, no major congenital anomalies and normal anthropometrics (**Table 2**).

**Table 2:** Anthropometric characteristics of enrolled newborns stratified by sex at T0 (135 (M 90)), compared with WHO 2010 Z-score.

		Overall	Males	Females	p-value
Weight (kg)	Mean (SD)	3.37 (0.34)	3.44 (0.32)	3.27 (0.35)	**0.004**
	Z-score (SD)	0.16 (0.71)	0.20 (0.65)	0.09 (0.79)	0.500
Length (cm)	Mean (SD)	50.00 (2.00)	51.00 (2.00)	50.00 (2.00)	**0.002**
	Z-score (SD)	0.21 (0.87)	0.24 (0.82)	0.17 (0.95)	0.300
Head circumference (cm)	Mean (SD)	34.37 (1.31)	34.49 (1.30)	34.17 (1.31)	0.072

Significant value are highlighted in bold (p>0.05).

At three months 109 infants were evaluated (64 males, 58.7%) and at 6 months 98 infants (60 males, 61.2%) with anthropometrics consistent with World Health Organization growth standards, indicating normal postnatal development.

### 3.2 Urinary hormones concentration

Urinary concentrations of key reproductive hormones measured during minipuberty (uLH, uFSH, uT in males, and uLH, uFSH and uE in females) in the population studied during the first six months of life were analyzed. Detailed values of hormones levels are provided in **Table 3** for males (upper panel) and females (lower panel). These data serve as the baseline for subsequent analyses exploring associations with urinary PAE metabolite concentrations.

**Table 3:** Urinary sex hormones levels at T0, T3, T6 in male and female infants.

Sex	Timepoint	Hormone	n	n<LOD (%)	Mean (SD)	Median (IQR)
Males	T0	uLH	89	28 (31.5)	0.64 (1.03)	0.27 (<LOD;0.62)
		uFSH	89	–	1.67 (1.39)	1.03 (0.79;1.98)
		uT	90	–	10.10 (5.20)	11.60 (4.9;15.00)
	T3	uLH	64	35 (54.7)	0.16 (0.20)	<LOD (<LOD;0.31)
		uFSH	64	–	0.90 (0.32)	0.84 (0.67;1.01)
		uT	63	–	0.43 (0.43)	0.28 (0.22;0.50)
	T6	uLH	59	30 (50)	0.16 (0.18)	0.14 (<LOD;0.30)
		uFSH	60	–	0.90 (0.56)	0.78 (0.62;1.04)
		uT	59	–	0.22 (0.17)	0.17 (0.13;0.26)
Females	T0	uLH	45	29 (64.4)	0.11 (0.21)	<LOD (<LOD;0.10)
		uFSH	45	–	1.34 (1.93)	0.70 (0.54;0.97)
		uE	45	–	789 (996)	403 (140,886)
	T3	uLH	45	26 (57.7)	0.11 (0.14)	<LOD (<LOD;0.25)
		uFSH	45	–	3.20 (3.23)	2.07 (1.58;3.03)
		uE	45	–	23 (10)	20 (15;25)
	T6	uLH	38	20 (52.6)	0.13 (0.15)	<LOD (<LOD;0.25)
		uFSH	38	–	3.64 (3.40)	2.29 (1.80;4.03)
		uE	37	–	24 (11)	22 (18;30)

uLH and uFSH are measured in mIU/mL while uT in ng/mL and uE in pg/mL. SD, standard deviation. IQR, interquartile range. LOD, Limit Of Detection.

### 3.3 Urinary phthalate levels

Urinary concentrations of the main PAE metabolites were measured to assess early-life exposure in infants. Metabolites of two or more PAEs resulted detectable in every sample, confirming the widespread perinatal and early postnatal exposure to PAEs among healthy term-born infants.

**Figure 2:**
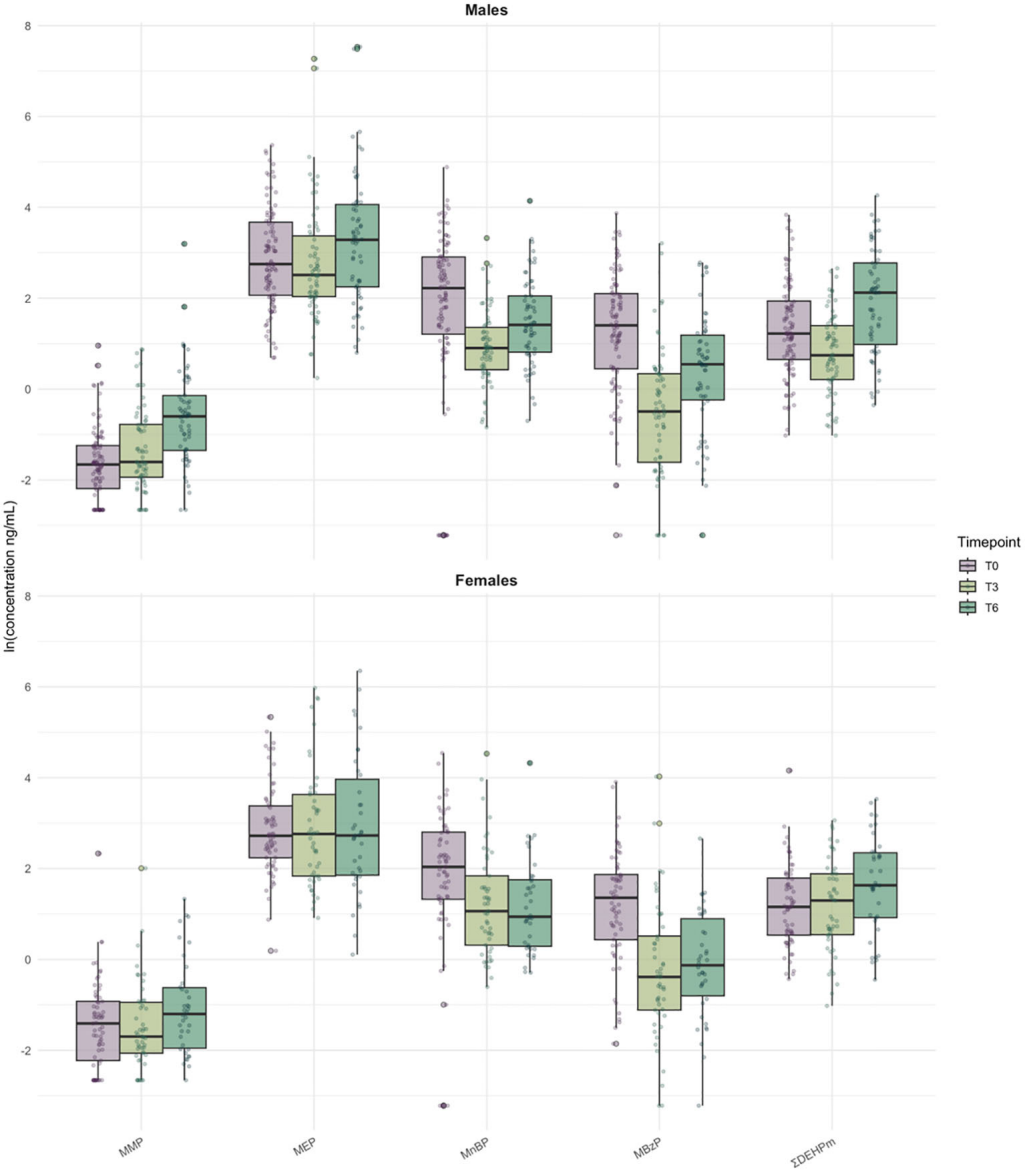
Distribution of urinary phthalate metabolite concentrations at birth (T0), 3 (T3), and six months (T6) in male and female infants. Box plots represent natural log-transformed concentrations of MMP, MEP, MnBP, MBzP, and ΣDEHPm. Male infants are shown in the upper panel and female infants in the lower panel. Boxes represent IQR, horizontal line indicates median, and whiskers indicate range.

The distribution of metabolite levels was skewed, ranging from low to moderately high levels (**Figure 2**; **Supplementary Table 3** in supplementary). Overall, at T3 infants exhibited the lowest concentrations for many PAE metabolites, whereas at T6 infants showed higher concentrations of most PAEs’ metabolites. At all timepoints and in both sexes MEP, the main metabolite of DEP, an unregulated PAE, showed the highest levels.

### 3.4 Association between urinary sex hormones values and phthalates within the first six months of life

**Figure 3:**
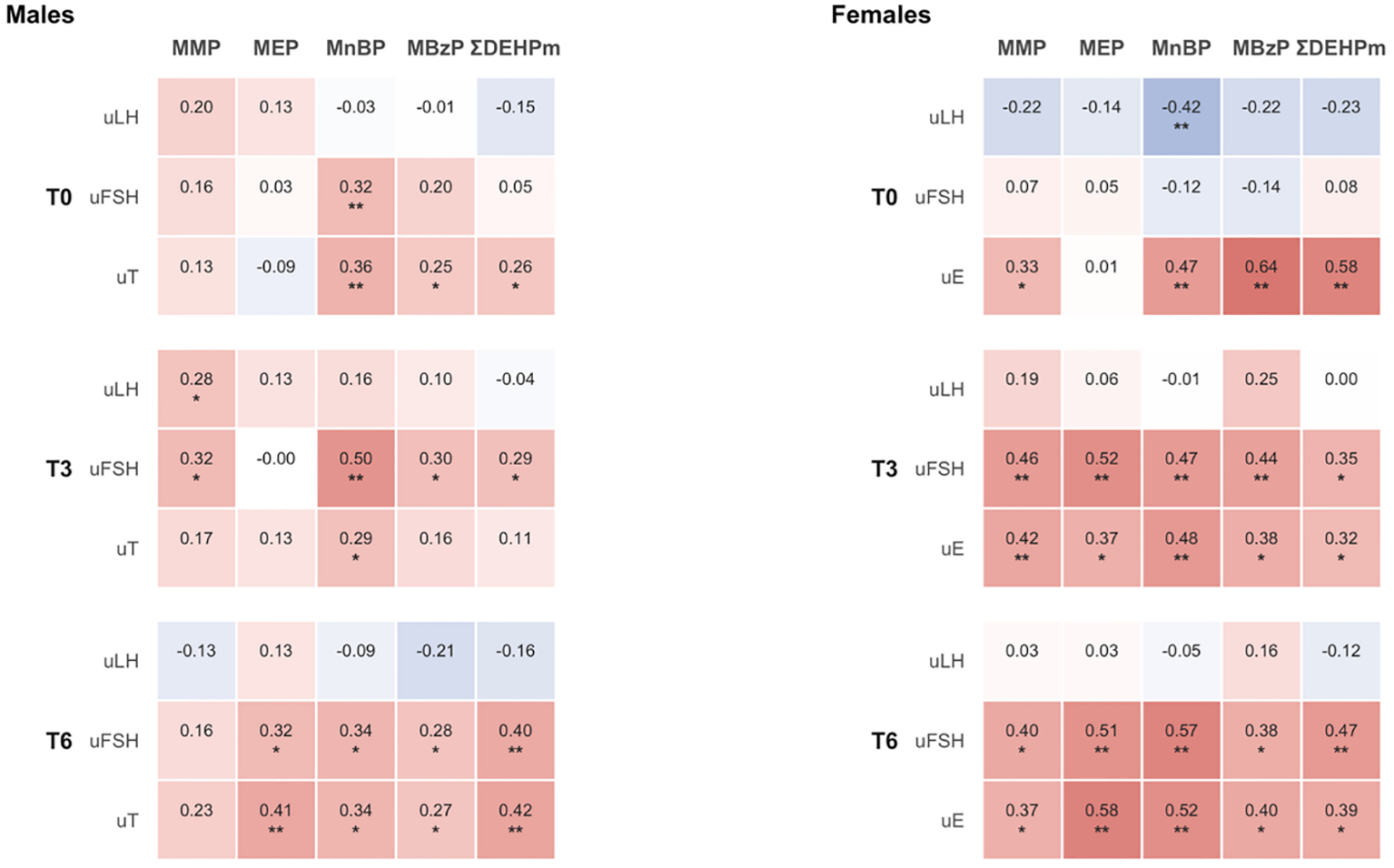
Heatmap of Spearman correlations between urinary phthalate metabolites and sex hormone levels at T0, T3, and T6 in male (left) and female (right) infants. Correlation coefficients were calculated separately by sex. Color scale ranges from −1 (dark blue, strong negative correlation) to +1 (dark red, strong positive correlation), with 0 (white) indicating no correlation. All concentrations were ln-transformed prior to analysis. Statistical significance is indicated as follows: *p < 0.05, **p < 0.005.

**Figure 4:**
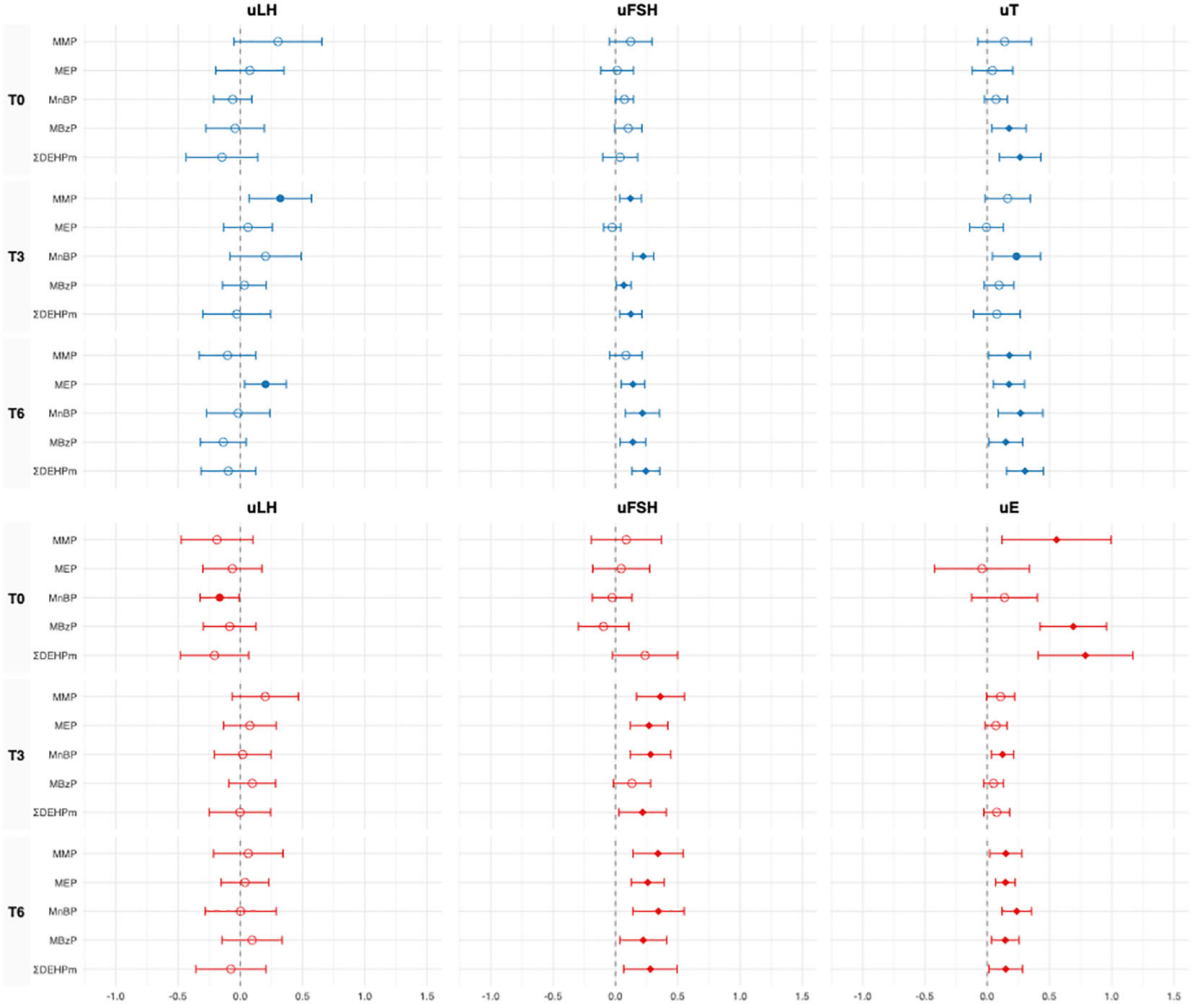
Forest plot of linear regression analyses between urinary phthalate metabolites and sex hormone levels at birth (T0), 3 (T3), and 6 months (T6) in male (upper panel) and female (lower pannel) infants. Outcomes include sex hormones levels evaluated at each timepoint. Points represent regression coefficients with 95% confidence intervals. All concentrations were ln-transformed prior to analysis. ˆp=NS, •p<0.05, ♦p(BH)<0.05.

We identified several associations between PAE concentrations and sex urinary hormonal profiles in both male and female infants throughout minipuberty as reported in **Figures 3**, **4**, **Tables 4**, **5**.

**Table 4:** Comparison of single-pollutant linear regression, Weighted Quantile Sum (WQS), and quantile g-computation (qgcomp) models assessing associations between phthalate metabolite exposure and urinary sex hormone levels in male infants at T0, T3, and T6.

Metabolite	T0					T3					T6				
	Linear regression		Mixture (weights)			Linear regression		Mixture (weights)			Linear regression		Mixture (weights)		
	β	p	W^+^	W^-^	qg	β	p	W^+^	W^-^	Qg	β	p	W^+^	W^-^	qg
uLH — N: T0 = 89, T3 = 64, T6 = 59															
MMP	0.303	0.092	0.248	0.161	0.480	**0.321**	**0.013**	0.623	0.004	0.480	-0.103	0.369	0.147	0.373	-0.236
MEP	0.076	0.580	0.209	0.235	0.012	0.062	0.529	0.275	0.054	0.052	**0.202**	**0.019**	0.426	0.087	0.729
MnBP	-0.061	0.430	0.127	0.064	0.049	0.203	0.161	0.032	0.214	0.468	-0.017	0.893	0.034	0.164	0.271
MBzP	-0.042	0.723	0.416	0.013	0.460	0.032	0.712	0.063	0.175	-0.134	-0.137	0.141	0.096	0.246	-0.592
ΣDEHPm	-0.148	0.311	0.001	0.528	-1.000	-0.028	0.837	0.008	0.553	-0.866	-0.096	0.385	0.297	0.130	-0.172
*Mix. β/ψ*			0.139	0.020	0.044			0.231	0.207	0.289			0.052	-0.181	-0.034
*p-value*			0.588	0.940	0.831			0.224	0.301	0.065			0.804	0.397	0.827
uFSH — N: T0 = 89, T3 = 64, T6 = 59															
MMP	0.122	0.162	0.117	0.390	0.120	**0.120**	**0.008**	0.413	0.016	0.295	0.084	0.208	0.003	0.451	-0.374
MEP	0.014	0.836	0.114	0.207	-0.248	-0.026	0.442	0.124	0.271	-0.620	**0.141**	**0.005**	0.007	0.383	0.131
MnBP	0.072	0.051	0.520	0.000	0.632	**0.222**	**<0.001**	0.400	0.000	0.705	**0.217**	**0.002**	0.008	0.000	-0.625
MBzP	0.103	0.069	0.202	0.077	0.248	**0.067**	**0.028**	0.002	0.594	-0.235	**0.140**	**0.009**	0.057	0.166	-0.001
ΣDEHPm	0.037	0.596	0.047	0.327	-0.752	**0.123**	**0.008**	0.060	0.120	-0.146	**0.244**	**<0.001**	0.924	0.000	0.869
*Mix. β/ψ*			**0.218**	0.104	0.154			**0.175**	**0.138**	**0.152**			**0.224**	**0.267**	**0.251**
*p-value*			**0.038**	0.371	0.089			**0.015**	**0.046**	**0.002**			**0.010**	**0.016**	**0.002**
uT — N: T0 = 90, T3 = 63, T6 = 58															
MMP	0.140	0.198	0.059	0.573	0.241	0.164	0.077	0.281	0.236	0.284	**0.178**	**0.038**	0.057	0.344	-0.253
MEP	0.043	0.608	0.100	0.258	-1.000	-0.005	0.939	0.091	0.302	-0.021	**0.175**	**0.007**	0.331	0.656	0.167
MnBP	0.071	0.129	0.413	0.000	0.482	**0.235**	**0.018**	0.566	0.002	0.716	**0.268**	**0.004**	0.163	0.000	-0.747
MBzP	**0.176**	**0.013**	0.242	0.063	0.031	0.096	0.112	0.048	0.164	-0.137	**0.149**	**0.033**	0.222	0.000	0.112
ΣDEHPm	**0.264**	**0.002**	0.186	0.106	0.247	0.078	0.411	0.014	0.296	-0.842	**0.303**	**<0.001**	0.226	0.000	0.721
*Mix. β/ψ*			**0.396**	0.197	**0.283**			0.278	0.215	0.211			0.257	0.109	**0.350**
*p-value*			**0.013**	0.211	**0.010**			0.057	0.178	0.074			0.052	0.414	**<0.001**

Results include association estimates from linear regression (β, p-values), WQS models (positive and negative indices; β, p-values, and mixture weights), and qgcomp models (ψ, p-values, and component weights for each metabolite). All analyses are stratified by timepoint, and all concentrations were ln-transformed prior to analysis. WQS and qgcomp weights represent the relative contribution of each metabolite within the mixture.

Significant value are highlighted in bold (p>0.05).

**Table 5:** Comparison of single-pollutant linear regression, Weighted Quantile Sum (WQS), and quantile g-computation (qgcomp) models assessing associations between phthalate metabolite exposure and urinary sex hormone levels in female infants at T0, T3, and T6.

Metabolite	T0					T3					T6				
	Linear regression		Mixture (weights)			Linear regression		Mixture (weights)			Linear regression		Mixture (weights)		
	β	p	W^+^	W^-^	qg	β	p	W^+^	W^-^	qg	β	p	W^+^	W^-^	qg
uLH															
MMP	-0.187	0.197	0.010	0.237	-0.167	0.200	0.136	0.157	0.089	0.226	0.064	0.644	0.045	0.417	-0.011
MEP	-0.064	0.588	0.112	0.103	-0.023	0.077	0.467	0.054	0.172	-0.095	0.038	0.691	0.447	0.078	0.065
MnBP	**-0.166**	**0.040**	0.045	0.406	-0.761	0.019	0.867	0.000	0.526	-0.716	0.002	0.988	0.012	0.295	-0.112
MBzP	-0.085	0.421	0.281	0.202	1.00	0.097	0.305	0.633	0.000	0.774	0.094	0.434	0.382	0.022	0.935
ΣDEHPm	-0.207	0.135	0.552	0.052	-0.049	-0.002	0.985	0.156	0.212	-0.189	-0.076	0.586	0.114	0.189	-0.878
*Mix. β/ψ*			-0.254	-0.263	-0.295			**0.460**	0.071	0.138			0.257	0.179	0.007
*p-value*			0.184	0.201	0.088			**0.046**	0.775	0.388			0.190	0.357	0.969
uFSH															
MMP	0.086	0.540	0.412	0.154	0.302	**0.361**	**<0.001**	0.134	0.171	0.186	**0.342**	**0.001**	0.048	—	-0.154
MEP	0.046	0.687	0.026	0.461	-0.050	**0.269**	**<0.001**	0.691	0.000	0.446	**0.259**	**<0.001**	0.289	—	0.239
MnBP	-0.027	0.738	0.045	0.084	-0.273	**0.282**	**0.001**	0.009	0.463	0.177	**0.345**	**0.002**	0.371	—	0.761
MBzP	-0.096	0.345	0.157	0.300	-0.677	0.132	0.082	0.093	0.366	0.079	**0.223**	**0.022**	0.052	—	-0.306
ΣDEHPm	0.238	0.074	0.359	0.001	0.698	**0.218**	**0.025**	0.072	0.000	0.113	**0.280**	**0.012**	0.240	—	-0.540
*Mix. β/ψ*			-0.101	0.007	0.008			0.297	0.426	0.399			0.249	—	**0.359**
*p-value*			0.538	0.970	0.955			0.098	0.032	0.002			0.083	—	**0.008**
uE															
MMP	**0.557**	**0.014**	0.325	0.000	0.294	0.108	0.059	0.252	0.000	-0.184	**0.150**	**0.024**	0.010	0.779	-0.328
MEP	-0.042	0.826	0.061	0.344	-0.408	0.070	0.117	0.330	0.000	0.116	**0.147**	**<0.001**	0.431	0.000	0.332
MnBP	0.140	0.292	0.012	0.457	-0.592	0.123	0.008	0.327	0.000	0.865	**0.237**	**<0.001**	0.362	0.000	0.592
MBzP	**0.692**	**<0.001**	0.378	0.092	0.488	0.051	0.202	0.029	0.794	0.019	**0.145**	**0.012**	0.156	0.050	0.077
ΣDEHPm	**0.789**	**<0.001**	0.224	0.107	0.218	0.077	0.139	0.062	0.206	-0.816	**0.149**	**0.031**	0.041	0.171	-0.672
*Mix. β/ψ*			**0.987**	**0.874**	**0.900**			0.125	0.085	0.115			**0.288**	**0.218**	**0.245**
*p-value*			**<0.001**	**0.003**	**<0.001**			0.304	0.420	**0.044**			**0.003**	**0.021**	**<0.001**

Results include association estimates from linear regression (β, p-values), WQS models (positive and negative indices; β, p-values, and mixture weights), and qgcomp models (ψ, p-values, and component weights for each metabolite). All analyses are stratified by timepoint, and all concentrations were ln-transformed prior to analysis. WQS and qgcomp weights represent the relative contribution of each metabolite within the mixture.

Significant value are highlighted in bold (p>0.05).

#### 3.4.1 Spearman correlation between urinary phthalates levels and urinary hormonal concentration in infants

As a first exploratory step, Spearman correlation coefficients were calculated to examine associations between each phthalate–hormone pair at each timepoint (T0, T3, T6), stratified by sex. This analysis was conducted prior to mixture analyses, allowing characterization of basic associations before evaluating combined exposure effects.

In male infants, Spearman correlations indicated generally positive associations between urinary PAE concentrations and urinary sex hormone levels across the first six months of life. uT showed weak to moderate correlations with MnBP at all timepoints and with ΣDEHPm at T0 and T6, while uFSH correlations tended to strengthen over time at T0 only with MnBP and at the other timepoints with all PAEs metabolites.

In female infants, similar patterns to males were observed at T3 and T6, while correlations at T0 were generally weak and not clinically significant. uE showed moderate positive correlations with MMP, MnBP, MBzP, and ΣDEHPm, a pattern that persisted at T3 and T6 for all metabolites. uLH displayed generally negative correlations at T0, with only one moderate significant correlation with MnBP. uFSH, as in males, showed moderate positive correlations with all metabolites at T3 and T6.

A comprehensive summary of correlation coefficients and p-values is presented in **Figure 3**.

#### 3.4.2 Linear regression analyses of urinary hormone levels on phthalate concentrations

Linear regression analyses were performed to assess the associations between each phthalate–hormone pair levels at each timepoint (T0, T3, and T6), stratified by sex, adjusted for age in day as well. The overall representation of the linear regression analysis is presented in **Figure 4**.

In male infants (**Table 4**; **Supplementary Table 4**), regression analyses indicated that exposure to PAE metabolites was significantly associated with uFSH levels at T3, particularly for MMP, MnBP, MBzP, and ΣDEHPm; these associations were confirmed at T6 for MEP, MnBP, MBzP, and ΣDEHPm. A similar pattern was observed for uT, with significant associations at T0 for MBzP and ΣDEHPm, and at T6 for all metabolites. Furthermore, at T3 no significant association was found with T/LH ratio.

In female infants (**Table 5**; **Supplementary Table 5**), broadly similar patterns were observed. Higher uFSH levels at T3 were associated with increased concentrations of MMP, MEP, and MnBP, while at T6 positive associations were observed across all metabolites. uE showed significant associations at T0 with MMP, MBzP, and ΣDEHPm, at T3 with MnBP, and at T6 with all metabolites.

For the above results, the associations remained significant after Benjamini–Hochberg correction.

#### 3.4.3 Associations between phthalate mixtures and urinary hormone levels using WQS regression and quantile g-computation

To further investigate the combined associations of PAE exposure, mixture analyses were performed using Weighted Quantile Sum (WQS) regression and quantile g-computation (qgcomp). These approaches allow the assessment of the joint effect of multiple correlated exposures, overcoming limitations of single-pollutant models. Consistent with previously reported analyses, both models were applied separately for each sampling time and for infant sex, to account for potential sex-specific differences, and were adjusted for age.

##### 3.4.3.1 Weighted quantile sum regression

In male infants (**Table 4**), at T0 exposure to increasing level of a mixture of PAEs appeared significantly positively associated with uFSH (β = 0.22, 95% CI: 0.02;0.42) and uT (β = 0.40, 95% CI: 0.09;0.70), with MnBP emerging as the metabolite with the highest statistical weight within the mixture for both hormones (weight=0.52 and 0.41, respectively) and MBzP also carrying a notable weight only for uT (weight=0.24).

At T3 and T6, results from mixture models remained significantly positively associated with uFSH (β = 0.18, 95% CI: 0.04;0.31; and β = 0.22, 95% CI: 0.07;0.38, respectively), with MMP (weight=0.41) and MnBP (weight=0.40) ranked among the highest-weighted components at T3, and ΣDEHPm (weight=0.92), accounting for the predominant share of the mixture index at T6.

In female infants (**Table 5**), associations were less consistent compared to males. AWQS index positively associated with uE was observed at T0 (β = 0.99, 95% CI: 0.54;1.44) and T6 (β = 0.29, 95% CI: 0.13;0.44), with MMP (weight=0.33), MBzP (weight=0.38), and ΣDEHPm (weight=0.22) representing the most heavily weighted components at T0, and MEP (weight=0.43) and MnBP (weight=0.36) carrying the largest proportional weights at T6.

Moreover, at T3, the WQS index was positively associated to uLH (β = 0.46, 95% CI: 0.04;0.88) with MBzP (weight=0.63) displaying the highest individual weight within the model.

##### 3.4.3.2 Quantile g-computation

In male infants (**Table 4**), index exposure to increasing level of a mixture of PAEs was positively associated with uT at T0 (ψ = 0.28, 95% CI: 0.07;0.49), with MnBP emerging as the highest-weighted component (weight=0.48).

At T3, the index remained positively associated with uFSH (ψ = 0.15, 95% CI: 0.06;0.24), with MnBP (weight=0.71) and MMP (weight=0.30) carrying the largest proportional weights within the model. At T6, the qgcomp index was positively associated with both uT (ψ = 0.35, 95% CI: 0.20;0.50) and uFSH (ψ = 0.25, 95% CI: 0.10;0.40), with ΣDEHPm accounting for the predominant share of the mixture index for both hormones (weight=0.87 and 0.72, respectively).

In female infants (**Table 5**), the qgcomp index was positively associated with uE at all time points: T0 (ψ = 0.90, 95% CI: 0.50;1.30), T3 (ψ = 0.12, 95% CI: 0.01;0.22), and T6 (ψ = 0.25, 95% CI: 0.12;0.37), with MBzP, ΣDEHPm and MMP ranking among the highest-weighted components at T0, MnBP and MEP at T3 and T6. Positive associations were also observed for uFSH at T3 (ψ = 0.40, 95% CI: 0.17;0.63) and T6 (ψ = 0.36, 95% CI: 0.11;0.61), with MEP and all metabolites carrying notable weights at T3 and MnBP and MEP emerging as the predominant components at T6.

Across all hormones, timepoints and sex strata with sufficient sample size (18 tests; **Supplementary Table 6**), only 2 quadratic terms reached nominal significance (minimum p = 0.034), consistent with chance expectation under the null and with none surviving correction for multiple testing.

#### 3.4.4 Comparison between linear regression, WQS and qgcomp results

Findings from all analytical approaches were consistent in indicating positive associations between exposure to increasing level of a mixture of PAEs and urinary sex hormone levels during the first six months of life, particularly for uFSH and uT in males and uFSH and uE in females.

Single-pollutant linear regression models identified significant associations with individual metabolites, highlighting MnBP, MBzP, and ΣDEHPm as recurrent predictors across time points. Mixture approaches (WQS and qgcomp) extended these results by capturing the joint effects of correlated exposures and quantifying the relative contribution of each metabolite within the mixture. A comprehensive comparison of results across analytical approaches is provided in the following tables, stratified by sex, with separate summaries reported for male (**Table 4**) and female (**Table 5**) infants.

Sex-specific patterns were highly consistent across methods. In male infants (**Table 4**), MnBP emerged as the main contributor to uFSH levels at T3 across all approaches, while ΣDEHPm predominated at T6, indicating a temporal shift in mixture composition.

In female infants (**Table 5**), mixture analyses indicated that multiple metabolites contributed to uE levels. At T0, MMP, MBzP, and ΣDEHPm jointly drove the association, suggesting a combined mixture association rather than a single dominant compound. At T6, the relative contribution shifted toward MEP and MnBP, supporting time-dependent changes in exposure relevance.

#### 3.4.5 Lagged analysis

Overall, lagged analyses showed sparse and confined to the shortest interval (T0 to T3) significant results, nevertheless, trends appeared consistent with the inverse concurrent associations reported in the main analysis. In single-pollutant models, levels of ΣDEHPm and MBzP at T0 were inversely associated with uLH in female infants at T3 (β = −0.39 and −0.26, respectively; *p*(BH) = 0.020 and 0.022), while the WQS and qgcomp mixture indices showed concordant but mostly non-significant trends. No statistically significant lagged associations were detected at the other time intervals (T0 to T6 and T3 to T6).

## 4 Discussion

In this longitudinal study, we investigated the association between early-life exposure to PAE metabolites and urinary sex hormone levels during minipuberty, a critical developmental window characterized by transient activation of the HPG-axis. Across complementary statistical approaches - including Spearman correlations, single-pollutant regression, WQS regression, and qgcomp - we observed consistent associations between phthalate exposure and hormonal variability during the first six months of life both in male and female. The observed urinary hormone concentrations were consistent with expected physiological ranges during minipuberty (52), the observed exposure to the different PAEs resulted as well in line with typical environmental exposure patterns, therefore we can reasonably interpret our findings within a relevant exposure context. As expected in an exploratory multi-exposure multi-outcome study, not all associations reached statistical significance across all strata; replication in larger prospective cohorts will be needed before conclusions about generalizability can be drawn.

Firstly, our finding suggests that there is a sex-specific pattern of associations. In males, PAE metabolites were consistently associated with uFSH and uT across timepoints, whereas in females the associations became more evident at later stages, particularly for uE and uFSH. These differences may reflect sex-specific HPG axis dynamics during minipuberty (31). In particular, the timing and intensity of HPG axis activation differ between sexes, with males exhibiting a more pronounced and early transient activation of gonadal steroidogenesis, whereas females show a more gradual and variable endocrine pattern during early infancy. In addition, sex-dependent differences in gonadal feedback regulation and tissue-specific sensitivity to gonadotropins may contribute to the observed temporal differences in PAE-hormone associations.

Across all models, MnBP, MBzP, and ΣDEHPm were repeatedly identified as metabolites with relatively higher statistical weights within the mixture models. This consistency across linear and mixture-based approaches supports the robustness of these statistical associations and suggests that these metabolites may warrant particular attention in future studies. Importantly, mixture models (WQS and qgcomp) extended single-pollutant analyses by capturing combined exposure effects, highlighting that PAE exposure acts as a complex mixture rather than as isolated compounds.

From a biological perspective, although the present study did not directly measure any mechanistic pathway, our findings are consistent with hypotheses drawn from the experimental literature suggesting that PAEs may interfere with multiple levels of the HPG axis including hypothalamic GnRH pulsatility, pituitary gonadotropin secretion, and gonadal steroidogenesis through PPAR-, AhR-, and estrogen receptor–mediated pathways (33). Based on experimental evidence, PAEs may interfere with kisspeptin/GnRH signaling, including IGF-1/PI3K/Akt/mTOR-dependent regulation of kisspeptin neurons and astrocyte-mediated modulation of GnRH secretion via prostaglandin E2 signaling, thereby altering gonadotropin output (33). These mechanistic pathways, while not directly assessed here, provide a speculative biological framework that is particularly relevant during minipuberty, when the HPG axis is transiently active and not yet fully stabilized by pubertal feedback mechanisms. In line with this, human evidence from the COPENHAGEN Minipuberty Study indicates that urinary concentrations of ∑DnBPm and ∑DEHPm are associated with altered LH, AMH, and testosterone/LH ratios in infant boys, suggesting early-life sensitivity of the HPG axis to PAE exposure, although these findings should be interpreted as associations rather than evidence of direct central disruption (35). Consistently, our observed positive associations with uFSH in both sexes may reflect upstream statistical patterns consistent with gonadal–pituitary feedback dysregulation during this developmental window.

At the gonadal level, experimental studies suggest that PAEs may impair steroidogenesis by downregulating key enzymes involved in testosterone and estradiol biosynthesis (e.g., StAR, CYP17A1, CYP19A1), inducing oxidative stress, and disrupting Sertoli and Leydig cell function through PPAR and AhR signaling pathways (33). Importantly, PAE-induced effects on steroid hormones may follow non-monotonic and biphasic dose–response relationships, particularly for DEHP, with differential effects observed across exposure levels due to complex receptor-mediated and feedback-regulated mechanisms (53). This non-linearity provides a biologically plausible framework to interpret the heterogeneous and time-dependent associations observed in the present study, including the varying contribution of individual metabolites within mixture models and the sex-specific patterns across timepoints. In this context, the observed increases in uT in male infants are consistent with, though do not directly demonstrate, transient statistical pattern potentially reflecting dysregulation of steroidogenic pathways during minipuberty rather than a simple linear response to gonadotropin stimulation, potentially reflecting developmental stage–dependent sensitivity of Leydig cell function and immature feedback regulation of the HPG axis.

Our results are also in line with experimental evidence suggesting that PAEs may exert time-dependent and context-dependent endocrine effects (54, 55). Depending on exposure level and developmental timing, PAEs have been reported to induce both stimulatory and inhibitory effects on steroidogenesis and gonadotropin signaling. These non-monotonic and dynamic responses, described in experimental models, may provide a speculative framework to interpret the heterogeneous patterns observed across timepoints in our cohort, particularly the increasing strength of associations at T3 and T6.

From a mixture toxicology perspective, the relatively higher statistical weights of MnBP and ΣDEHPm emerging consistently across models suggest these metabolites may deserve priority attention in future research, though their role as biological drivers of endocrine disruption cannot be established from the present observational data. The consistency between WQS and qgcomp results further supports this statistical pattern and highlights the importance of addressing environmental exposures as mixtures rather than single chemicals. Notably, both DEHP and DnBP (of which MnBP is a primary metabolite) are regulated under European Union legislation due to their recognized reproductive toxicity and endocrine-disrupting properties. DEHP is classified as toxic to reproduction (H360FD) with evidence of potential damage to fertility and the unborn child, while DnBP is classified as toxic for reproduction (H360Df), with concerns for developmental and fertility effects. Consequently, these substances are subject to restrictions in consumer products, including food contact materials, toys and childcare articles, cosmetic products, and are further restricted in medical devices (5). Their persistent detection, even though at lower levels than in past times, and their statistical prominence in our mixture models underscore the relevant role still played by regulatory priority substances in shaping real-world early-life exposure patterns.

This study has several strengths, including its longitudinal design, repeated urinary hormone and PAE metabolites measurements, and the use of complementary statistical approaches for mixture and single-exposure analyses. However, limitations should be acknowledged. Urinary PAE metabolites reflect short-term exposure and may not fully capture long-term exposure patterns. The primary cross-sectional analyses are based on concurrent measurements of PAE metabolites and reproductive hormones within the same urine sample at each timepoint. While reverse causation is biologically implausible, as urinary PAE metabolite concentrations reflect exogenous environmental exposure and cannot be influenced by the infant’s endocrine status, concurrent measurement does not allow formal conclusions about temporal precedence within the same timepoint, and all associations should be interpreted as concurrent statistical relationships. In addition, although multiple models and correction approaches were used, residual confounding cannot be excluded. No mechanistic pathways were directly assessed, and all biological interpretations should be considered speculative and hypothesis-generating, requiring confirmation in dedicated experimental and larger epidemiological studies. Finally, the absence of effect size estimates and formal matrix validation for urinary hormone assays represent additional methodological limitations that future studies should address.

In conclusion, our findings are consistent with the hypothesis that early-life exposure to PAE mixtures is associated with sex-specific differences in urinary sex hormone levels during minipuberty, a critical and highly sensitive window for the maturation of the reproductive axis. During this period, observed hormonal associations may reflect underlying HPG axis sensitivity to environmental exposures, with hypothetical long-term implications for fertility and pubertal development that warrant investigation in future longitudinal studies. The consistency across analytical approaches and the identification of MnBP, MBzP, and ΣDEHPm as metabolites with relatively higher mixture weights underscore the relevance of mixture-based approaches in endocrine epidemiology and highlight minipuberty as a sensitive window for environmental exposure.

## Data Availability

The raw data supporting the conclusions of this article will be made available by the authors, without undue reservation.
