# Supplementary material for: Early-life phthalate mixture exposure and reproductive hormone levels during minipuberty
Source: Front Endocrinol (Lausanne). 2026 Jul 22;17:1866609. doi: 10.3389/fendo.2026.1866609 (PMC13437279; doi:10.3389/fendo.2026.1866609)
Supplement: Supplementary file 1 [file Supplementaryfile1.docx]

Supplementary Material

# 1 Supplementary Data

**Supplementary information for phthalate analytical procedure**

Phthalate analytical procedure: List of mail reagents and standards

- Acetonitrile, Fisher Scientific (Milano, Italy)
- Methanol, Fisher Scientific (Milano, Italy)
- Fosforic Acid Carlo Erba (Milano, Italy)
- Glacial Acetic acetic, Carlo Erba (Milano, Italy)
- Ammonium Acetate, Carlo Erba (Milano, Italy).
- β -glucuronidase (Escherichia coli K12), Roche Diagnostics (Mannheim, Germany).
- MMP - Monomethyl phthalate 100 μg/mL in Acetonitrile 1 ml, Restek (Milano, Italy)
- MEP - Monoethyl phthalate 100 μg/mL in Acetonitrile 1 mL, Labservice Analytica (Bologna, Italy)
- MnBP - Mono-n-butyl phthalate 100 μg/mL in Acetonitrile 1 mL, Labservice Analytica (Bologna, Italy)
- MBzP - Monobenzyl phthalate 100 μg/mL in Acetonitrile 1 mL, Labservice Analytica (Bologna, Italy)
- MEHP - Mono(2-ethylhexyl) phthalate 100 μg/mL in Acetonitrile 1 ml, Labservice Analytica (Bologna, Italy)
- MEHHP – mono-(2-ethyl-5-hydroxyhexyl) phthalate 100 μg/mL in MTBE, Labservice Analytica (Bologna, Italy)
- MEOHP - mono-(2-ethyl-5-oxohexyl) phthalate 100 μg/mL in MTBE, Labservice Analytica (Bologna, Italy)
- Monomethyl Phthalate 100 μg/mL in Acetonitrile (Ring-1,2-13C 2, DICARBOXYL-13C2,99%) 1,2 mL MTBE Labservice Analytica (Bologna, Italy)
- Monoethyl Phthalate 100 μg/mL in Acetonitrile (RING-1,2-13C 2, DICARBOXYL- 13C2,99%) 1,2 mL MTBE Labservice Analytica (Bologna, Italy)

# 2 Supplementary Figures and Tables

## 2.1 Supplementary Tables

|  | **LOD**  **ppb** | **LOQ**  **ppb** | **Correlation Coefficient** | **Absolute % recovery** | **Linearity range**  **ppb** |
| --- | --- | --- | --- | --- | --- |
| **MMP** | 0,1 | 0,25 | 0.9994 | 53.5 | 0 - 250 |
| **MEP** | 0,1 | 0,25 | 0.9995 | 94.0 | 0 - 250 |
| **MEHHP** | 0,05 | 0,1 | 0.9991 | 80.1 | 0 - 250 |
| **MEOHP** | 0,05 | 0,1 | 0.9998 | 98.6 | 0 - 250 |
| **MNBP** | 0,05 | 0,1 | 0.9994 | 70.0 | 0 - 250 |
| **MBzP** | 0,05 | 0,1 | 0.9995 | 99.4 | 0 - 250 |
| **MEHP** | 0,25 | 0,5 | 0.9990 | 82.2 | 0 - 250 |

Table S1 - *Phthalate analytical procedure: main analytical methodology validation parameters*

| **Step** | **Time (min)** | **Flow mL/min** | **% mobile phase A** | **% mobile phase B** |
| --- | --- | --- | --- | --- |
| **1** | 0 | 0.3 | 98 | 2 |
| **2** | 0.5 | 0.3 | 92 | 8 |
| **3** | 25.5 | 0.3 | 2 | 98 |
| **4** | 29.9 | 0.3 | 2 | 98 |
| **5** | 30 | 0.3 | 92 | 8 |

Table S2 - *Phthalate analytical procedure: UHPL/MS flow rate and solvent gradients*

| **Sex** | **Timepoint** | **PAEs** | **n** | **n<LOD (%)** | **Mean** | **DS** | **Median** | **Q1** | **Q3** | **Min** | **Max** |
| --- | --- | --- | --- | --- | --- | --- | --- | --- | --- | --- | --- |
| **Males** | **T0** | **MMP** | 90 | 19 (21.1) | 0.29 | 0.37 | 0.19 | 0.11 | 0.29 | 0.07 | 2.60 |
|  |  | **MEP** | 90 | - | 32.33 | 40.86 | 15.91 | 7.78 | 38.09 | 1.99 | 215.06 |
|  |  | **MnBP** | 90 | 8 (8.9) | 15.34 | 19.22 | 8.91 | 3.66 | 18.59 | 0.04 | 132.27 |
|  |  | **MBzP** | 90 | 1 (1.1) | 6.87 | 8.00 | 4.07 | 1.61 | 8.20 | 0.04 | 47.94 |
|  |  | **ΣDEHPm** | 90 | - | 6.79 | 8.57 | 3.46 | 2.13 | 7.49 | 0.41 | 46.24 |
|  | **T3** | **MMP** | 64 | 6 (9.4) | 0.44 | 0.55 | 0.20 | 0.14 | 0.47 | 0.07 | 2.40 |
|  |  | **MEP** | 64 | - | 62.47 | 227.58 | 12.22 | 7.63 | 23.85 | 1.28 | 1,438.62 |
|  |  | **MnBP** | 64 | - | 3.82 | 4.45 | 2.48 | 1.49 | 4.04 | 0.43 | 27.75 |
|  |  | **MBzP** | 64 | 5 (7.8) | 1.74 | 4.00 | 0.64 | 0.21 | 1.41 | 0.04 | 24.76 |
|  |  | **ΣDEHPm** | 64 | - | 3.28 | 3.12 | 2.00 | 1.22 | 4.13 | 0.36 | 14.25 |
|  | **T6** | **MMP** | 60 | 2 (3.3) | 1.19 | 3.20 | 0.54 | 0.25 | 0.94 | 0.07 | 24.44 |
|  |  | **MEP** | 60 | - | 103.32 | 328.11 | 26.73 | 9.65 | 57.29 | 2.23 | 1,868.98 |
|  |  | **MnBP** | 60 | - | 7.34 | 9.47 | 4.25 | 2.37 | 8.17 | 0.50 | 62.96 |
|  |  | **MBzP** | 60 | 1 (1,7) | 3.10 | 4.10 | 1.76 | 0.82 | 3.42 | 0.04 | 16.20 |
|  |  | **ΣDEHPm** | 60 | - | 12.28 | 13.77 | 8.35 | 2.88 | 15.98 | 0.70 | 71.02 |
|  | | | | | | | | | | | |
| **Females** | **T0** | **MMP** | 45 | 10 (22.2) | 0.32 | 0.28 | 0.22 | 0.10 | 0.43 | 0.07 | 1.47 |
|  |  | **MEP** | 45 | - | 29.87 | 40.50 | 15.21 | 8.57 | 33.61 | 1.21 | 207.69 |
|  |  | **MnBP** | 45 | 2 (4.4) | 13.04 | 16.28 | 6.46 | 3.84 | 16.61 | 0.04 | 93.92 |
|  |  | **MBzP** | 45 | - | 5.90 | 7.52 | 4.01 | 1.55 | 7.41 | 0.22 | 44.42 |
|  |  | **ΣDEHPm** | 45 | - | 5.52 | 9.51 | 3.24 | 1.71 | 5.87 | 0.65 | 63.98 |
|  | **T3** | **MMP** | 45 | 7 (15.6) | 0.46 | 1.12 | 0.18 | 0.12 | 0.36 | 0.07 | 7.43 |
|  |  | **MEP** | 45 | - | 43.38 | 86.07 | 12.92 | 6.24 | 32.70 | 2.49 | 393.40 |
|  |  | **MnBP** | 45 | - | 8.04 | 16.09 | 2.73 | 1.27 | 6.30 | 0.55 | 92.72 |
|  |  | **MBzP** | 45 | 2 (4.4) | 2.98 | 8.73 | 0.68 | 0.33 | 1.67 | 0.04 | 56.07 |
|  |  | **ΣDEHPm** | 45 | - | 5.36 | 5.30 | 3.71 | 1.68 | 6.93 | 0.36 | 21.38 |
|  | **T6** | **MMP** | 38 | 1 (2.6) | 0.63 | 0.86 | 0.30 | 0.14 | 0.54 | 0.07 | 3.79 |
|  |  | **MEP** | 38 | - | 61.10 | 116.68 | 15.31 | 6.39 | 53.46 | 1.11 | 574.04 |
|  |  | **MnBP** | 38 | - | 6.29 | 12.28 | 2.56 | 1.34 | 5.78 | 0.75 | 75.50 |
|  |  | **MBzP** | 38 | 1 (2.6) | 1.70 | 2.45 | 0.88 | 0.45 | 2.48 | 0.04 | 14.31 |
|  |  | **ΣDEHPm** | 38 | - | 8.56 | 8.53 | 5.12 | 2.51 | 10.47 | 0.64 | 34.13 |

Table S3 - *Levels of urinary phthalate metabolite concentrations (ng/mL) at birth (T0), 3 (T3), and six months (T6) in male and female infants. Box plots represent natural log-transformed concentrations of MMP, MEP, MnBP, MBzP, and ΣDEHPm. Male infants are shown in the upper panel and female infants in the lower panel. DS: Deviation Standard; Q1:First Quartile; Q3: Third Quartile; min: minimum; max:maximum.*

|  | Hormone | PAEs | n | β (95% CI) | *p* | *p*(BH) |
| --- | --- | --- | --- | --- | --- | --- |
| **T0** | **uLH** | **MMP** | 89 | 0.30 (−0.05; 0.66) | 0.092 | 0.462 |
|  |  | **MEP** | 89 | 0.08 (−0.20; 0.35) | 0.580 | 0.723 |
|  |  | **MnBP** | 89 | −0.06 (−0.21; 0.09) | 0.430 | 0.717 |
|  |  | **MBzP** | 89 | −0.04 (−0.28; 0.19) | 0.723 | 0.723 |
|  |  | **ΣDEHPm** | 89 | −0.15 (−0.44; 0.14) | 0.311 | 0.717 |
|  | **uFSH** | **MMP** | 89 | 0.12 (−0.05; 0.29) | 0.162 | 0.270 |
|  |  | **MEP** | 89 | 0.01 (−0.12; 0.14) | 0.836 | 0.836 |
|  |  | **MnBP** | 89 | 0.07 (−0.00; 0.15) | 0.051 | 0.172 |
|  |  | **MBzP** | 89 | 0.10 (−0.01; 0.21) | 0.069 | 0.172 |
|  |  | **ΣDEHPm** | 89 | 0.04 (−0.10; 0.18) | 0.596 | 0.745 |
|  | **uT** | **MMP** | 90 | 0.14 (−0.08; 0.36) | 0.198 | 0.248 |
|  |  | **MEP** | 90 | 0.04 (−0.12; 0.21) | 0.608 | 0.608 |
|  |  | **MnBP** | 90 | 0.07 (−0.02; 0.16) | 0.129 | 0.216 |
|  |  | **MBzP** | 90 | 0.18 (0.04; 0.31) | **0.013** | **0.032** |
|  |  | **ΣDEHPm** | 90 | 0.26 (0.10; 0.43) | **0.002** | **0.012** |
| **T3** | **uLH** | **MMP** | 64 | 0.32 (0.07; 0.57) | **0.013** | 0.066 |
|  |  | **MEP** | 64 | 0.06 (−0.13; 0.53) | 0.529 | 0.837 |
|  |  | **MnBP** | 64 | 0.20 (−0.08; 0.49) | 0.161 | 0.402 |
|  |  | **MBzP** | 64 | 0.03 (−0.14; 0.21) | 0.712 | 0.837 |
|  |  | **ΣDEHPm** | 64 | −0.03 (−0.30; 0.24) | 0.837 | 0.837 |
|  | **uFSH** | **MMP** | 64 | 0.12 (0.03; 0.21) | **0.008** | **0.014** |
|  |  | **MEP** | 64 | −0.03 (−0.10; 0.04) | 0.442 | 0.442 |
|  |  | **MnBP** | 64 | 0.22 (0.14; 0.31) | **<0.001** | **<0.001** |
|  |  | **MBzP** | 64 | 0.07 (0.01; 0.13) | **0.028** | **0.035** |
|  |  | **ΣDEHPm** | 64 | 0.12 (0.03; 0.21) | **0.008** | **0.014** |
|  | **uT** | **MMP** | 63 | 0.16 (−0.02; 0.35) | 0.077 | 0.187 |
|  |  | **MEP** | 63 | −0.01 (−0.14; 0.13) | 0.939 | 0.939 |
|  |  | **MnBP** | 63 | 0.24 (0.04; 0.43) | **0.018** | 0.089 |
|  |  | **MBzP** | 63 | 0.10 (−0.02; 0.22) | 0.112 | 0.187 |
|  |  | **ΣDEHPm** | 63 | 0.08 (−0.11; 0.27) | 0.411 | 0.513 |
| **T6** | **uLH** | **MMP** | 59 | −0.10 (−0.33; 0.13) | 0.369 | 0.481 |
|  |  | **MEP** | 59 | 0.20 (0.04; 0.37) | **0.019** | 0.093 |
|  |  | **MnBP** | 59 | −0.02 (−0.27; 0.24) | 0.893 | 0.893 |
|  |  | **MBzP** | 59 | −0.14 (−0.32; 0.05) | 0.141 | 0.352 |
|  |  | **ΣDEHPm** | 59 | −0.10 (−0.32; 0.12) | 0.385 | 0.481 |
|  | **uFSH** | **MMP** | 59 | 0.08 (−0.05; 0.22) | 0.208 | 0.208 |
|  |  | **MEP** | 59 | 0.14 (0.05; 0.24) | **0.005** | **0.008** |
|  |  | **MnBP** | 59 | 0.22 (0.08; 0.35) | **0.002** | **0.006** |
|  |  | **MBzP** | 59 | 0.14 (0.04; 0.24) | **0.009** | **0.011** |
|  |  | **ΣDEHPm** | 59 | 0.24 (0.13; 0.36) | **<0.001** | **<0.001** |
|  | **uT** | **MMP** | 58 | 0.18 (0.01; 0.35) | **0.038** | **0.038** |
|  |  | **MEP** | 58 | 0.18 (0.05; 0.30) | **0.007** | **0.012** |
|  |  | **MnBP** | 58 | 0.27 (0.09; 0.45) | **0.004** | **0.010** |
|  |  | **MBzP** | 58 | 0.15 (0.01; 0.29) | **0.033** | **0.038** |
|  |  | **ΣDEHPm** | 58 | 0.30 (0.16; 0.45) | **<0.001** | **<0.001** |

Table S4 - *Linear regression analyses of associations between urinary phthalate metabolite concentrations and sex hormone levels in female infants at T0, T3, and T6.*

*Regression coefficients (β), 95% confidence intervals (95% CI), p-values, and p-value after Benjamini‒Hochberg correction (p(BH) are reported for the associations between individual phthalate metabolites (PAEs) and urinary hormone levels. Analyses were conducted separately at each timepoint (T0, T3, T6).*

*Statistically significant associations are highlighted in bold.*

|  | Hormone | PAEs | n | β (95% CI) | *p* | *p*(BH) |
| --- | --- | --- | --- | --- | --- | --- |
| **T0** | **uLH** | **MMP** | 45 | −0.19 (−0.48;0.10) | 0.197 | 0.328 |
|  |  | **MEP** | 45 | −0.06 (−0.30;0.17) | 0.5885 | 0.5885 |
|  |  | **MnBP** | 45 | −0.1661 (−0.32; −0.01) | **0.040** | 0.200 |
|  |  | **MBzP** | 45 | −0.09 (−0.30;0.13) | 0.421 | 0.526 |
|  |  | **ΣDEHPm** | 45 | −0.21 (−0.48;0.07) | 0.135 | 0.328 |
|  | **uFSH** | **MMP** | 45 | 0.09 (−0.20;0.37) | 0.540 | 0.739 |
|  |  | **MEP** | 45 | 0.05 (−0.18;0.28) | 0.687 | 0.739 |
|  |  | **MnBP** | 45 | −0.03 (−0.19;0.13) | 0.739 | 0.739 |
|  |  | **MBzP** | 45 | −0.10 (−0.30;0.11) | 0.345 | 0.739 |
|  |  | **ΣDEHPm** | 45 | 0.24 (−0.02;0.50) | 0.074 | 0.368 |
|  | **uE** | **MMP** | 45 | 0.56 (0.12;1.00) | **0.014** | **0.024** |
|  |  | **MEP** | 45 | −0.04 (−0.42;0.34) | 0.826 | 0.826 |
|  |  | **MnBP** | 45 | 0.14 (−0.13;0.40) | 0.292 | 0.366 |
|  |  | **MBzP** | 45 | 0.69 (0.43;0.96) | **<0.001** | **<0.001** |
|  |  | **ΣDEHPm** | 45 | 0.79 (0.41;1.17) | **<0.001** | **<0.001** |
| **T3** | **uLH** | **MMP** | 45 | 0.20 (−0.07;0.47) | 0.136 | 0.679 |
|  |  | **MEP** | 45 | 0.08 (−0.13;0.29) | 0.467 | 0.778 |
|  |  | **MnBP** | 45 | 0.02 (−0.21;0.25) | 0.867 | 0.985 |
|  |  | **MBzP** | 45 | 0.10 (−0.09;0.28) | 0.305 | 0.762 |
|  |  | **ΣDEHPm** | 45 | −0.00 (−0.25;0.24) | 0.985 | 0.985 |
|  | **uFSH** | **MMP** | 45 | 0.36 (0.17;0.55) | **<0.001** | **0.002** |
|  |  | **MEP** | 45 | 0.27 (0.12;0.42) | **0.001** | **0.002** |
|  |  | **MnBP** | 45 | 0.28 (0.12;0.45) | **0.001** | **0.002** |
|  |  | **MBzP** | 45 | 0.13 (−0.02;0.28) | 0.082 | 0.082 |
|  |  | **ΣDEHPm** | 45 | 0.22 (0.03;0.41) | 0.025 | 0.032 |
|  | **uE** | **MMP** | 45 | 0.11 (−0.01;0.22) | 0.060 | 0.149 |
|  |  | **MEP** | 45 | 0.07 (−0.02;0.16) | 0.117 | 0.174 |
|  |  | **MnBP** | 45 | 0.12 (0.03;0.21) | **0.008** | **0.041** |
|  |  | **MBzP** | 45 | 0.05 (−0.03;0.13) | 0.202 | 0.202 |
|  |  | **ΣDEHPm** | 45 | 0.08 (−0.03;0.18) | 0.139 | 0.174 |
| **T6** | **uLH** | **MMP** | 38 | 0.06 (−0.22;0.34) | 0.645 | 0.864 |
|  |  | **MEP** | 38 | 0.04 (−0.15;0.23) | 0.691 | 0.864 |
|  |  | **MnBP** | 38 | 0.00 (−0.28;0.34) | 0.988 | 0.988 |
|  |  | **MBzP** | 38 | 0.09 (−0.15;0.34) | 0.434 | 0.864 |
|  |  | **ΣDEHPm** | 38 | −0.08 (−0.36;0.21) | 0.586 | 0.864 |
|  | **uFSH** | **MEP** | 38 | 0.26 (0.13;0.39) | **<0.001** | **0.002** |
|  |  | **MMP** | 38 | 0.34 (0.14;0.54) | **0.002** | **0.003** |
|  |  | **MnBP** | 38 | 0.35 (0.14;0.55) | **0.002** | **0.003** |
|  |  | **MBzP** | 38 | 0.22 (0.04;0.41) | **0.022** | **0.022** |
|  |  | **ΣDEHPm** | 38 | 0.28 (0.07;0.50) | **0.012** | **0.015** |
|  | **uE** | **MMP** | 37 | 0.15 (0.02;0.28) | **0.024** | **0.030** |
|  |  | **MEP** | 37 | 0.15 (0.07;0.23) | **0.001** | **0.002** |
|  |  | **MnBP** | 37 | 0.24 (0.12;0.36) | **<0.001** | **0.001** |
|  |  | **MBzP** | 37 | 0.15 (0.03;0.26) | **0.012** | **0.021** |
|  |  | **ΣDEHPm** | 37 | 0.15 (0.01;0.28) | **0.031** | **0.031** |

Table S5 - *Linear regression analyses of associations between urinary phthalate metabolite concentrations and sex hormone levels in female infants at T0, T3, and T6.*

*Regression coefficients (β), 95% confidence intervals (95% CI), p-values, and p-value after Benjamini‒Hochberg correction (p(BH) are reported for the associations between individual phthalate metabolites (PAEs) and urinary hormone levels. Analyses were conducted separately at each timepoint (T0, T3, T6).*

*Statistically significant associations are highlighted in bold.*

| **Sex** | **Timepoint** | **Hormone** | **qgcomp (degree=1)** | | | **qgcomp (degree=2)** | | |
| --- | --- | --- | --- | --- | --- | --- | --- | --- |
|  |  |  | **𝜓_1_** | **95% CI** | ***p*** | **𝜓_2_** | **95% CI** | ***p*** |
| **Male** | **T0** | **uLH** | 0.04 | -0.36;0.44 | 0.831 | 0.258 | -0.26;0.77 | 0.327 |
|  |  | **uFSH** | 0.15 | -0.02;0.33 | 0.089 | -0.048 | -0.31;0.21 | 0.720 |
|  |  | **uT** | **0.28** | **0.07;0.49** | **0.010** | -0.043 | -0.30;0.21 | 0.744 |
|  | **T3** | **uLH** | 0.29 | -0.01;0.59 | 0.065 | 0.233 | -0.21;0.67 | 0.304 |
|  |  | **uFSH** | **0.15** | **0.06;0.24** | **0.002** | 0.102 | -0.05;0.25 | 0.194 |
|  |  | **uT** | 0.21 | -0.02;0.44 | 0.074 | -0.173 | -0.65;0.30 | 0.477 |
|  | **T6** | **uLH** | −0.03 | -0.34;0.27 | 0.827 | 0.420 | 0.00;0.84 | 0.054 |
|  |  | **uFSH** | **0.25** | **0.10;0.40** | **0.002** | 0.044 | -0.20;0.29 | 0.729 |
|  |  | **uT** | **0.35** | **0.20;0.50** | **<0.001** | 0.221 | -0.04;0.48 | 0.098 |
|  | | | | | | | | |
| **Female** | **T0** | **uLH** | −0.30 | -0.62;0.04 | 0.088 | -0.235 | -0.94;0.47 | 0.516 |
|  |  | **uFSH** | 0.01 | -0,25;0.27 | 0.955 | -0.121 | -0.71;0.47 | 0.693 |
|  |  | **uE** | **0.90** | **0.50;1.30** | **<0.001** | -0.148 | -1.01;0.71 | 0.738 |
|  | **T3** | **uLH** | 0.14 | -0.17;0.45 | 0.388 | -0.033 | -0.51;0.44 | 0.893 |
|  |  | **uFSH** | **0.40** | **0.17;0.63** | **0.002** | 0.331 | 0.03;0.63 | **0.041** |
|  |  | **uE** | **0.12** | **0.01;0.22** | **0.044** | 0.034 | -0.18;0.25 | 0.754 |
|  | **T6** | **uLH** | 0.01 | -0.37;0.38 | 0.969 | 0.431 | -0.25;1.11 | 0.226 |
|  |  | **uFSH** | **0.36** | **0.11;0.61** | **0.008** | 0.194 | -0.31;0.70 | 0.456 |
|  |  | **uE** | **0.25** | **0.12;0.37** | **<0.001** | 0.254 | 0.03;0.47 | **0.034** |

**Table S6 -** *Test for non-linearity of the mixture dose–response using quadratic qgcomp.*

*Degree-1 (linear) and degree-2 (quadratic) qgcomp models were fitted separately for each hormone, timepoint, and sex stratum. The degree-1 estimate (ψ₁) represents the linear mixture association; the degree-2 term (ψ₂) quantifies departure from linearity (curvature). Values in bold indicate statistical significance (p < 0.05). Upper panel: male infants; lower panel: female infants. T0, birth; T3, 3 months of age; T6, 6 months of age. uFSH, urinary follicle-stimulating hormone; uLH, urinary luteinizing hormone; uT, urinary testosterone; uE, urinary estradiol.*
